# Xanthogranulomatous Pyelonephritis Presenting as Significant Hydronephrosis in an Adult Male Who Was Managed with Robotic‐Assisted Radical Nephrectomy: A Case Report and Current Literature Review

**DOI:** 10.1155/criu/2534184

**Published:** 2026-03-10

**Authors:** Robert W. Jarrett Berends, Christopher Pulford, Charla Miller, Emiley O’Pry, Richard Eames, Kenneth Shockley, Thomas Maatman

**Affiliations:** ^1^ Department of Urological Surgery, University of Michigan Health-West, Wyoming, Michigan, USA

## Abstract

Xanthogranulomatous pyelonephritis (XGP) is an infection of the kidney that often results from a chronic obstruction, such as a kidney stone. We present a patient with a massively enlarged left renal pelvis with minimal renal parenchyma presenting as an end stage ureteropelvic junction obstruction (UPJO). The patient was afebrile during his time in the emergency room (ER). Urinalysis was significant for trace leukocyte esterase and 11–20 WBCs. His creatinine during his ER encounter was 1.02. Due to his normal creatinine, a percutaneous nephrostomy tube was not placed. Preoperatively, we did not suspect XGP or pyonephrosis given his prior ER results. Shortly after the patient′s ER presentation he was taken to surgery and final pathology revealed XGP. We were able to successfully perform a robotic‐assisted laparoscopic radical nephrectomy for stage I XGP safely, which included drainage of over two liters of pyogenic urine during the surgery.

## 1. Introduction

Xanthogranulomatous pyelonephritis (XGP) is an uncommon form of chronic pyelonephritis characterized by granulomatous inflammation and progressive destruction of the renal parenchyma [1–3]. It most commonly presents in women between 45 and 55 years of age. The typical etiology involves chronic urinary tract obstruction with superimposed infection. XGP may present as a urological emergency and, if left untreated, can extend beyond Gerota′s fascia into adjacent structures, often mimicking a malignant process [1–3]. As a result, early diagnosis is challenging yet critical to avoid delays in definitive management.

We report a case of XGP with an atypical presentation in which the patient was neither septic nor systemically ill and presented solely with renal colic. Imaging findings were suggestive of ureteropelvic junction obstruction (UPJO) rather than inflammatory disease. The patient ultimately underwent robotic‐assisted laparoscopic radical nephrectomy, with final pathology confirming XGP.

## 2. Case Presentation

The patient reported abdominal swelling and discomfort that had been present for approximately 2–3 months. Earlier in the year, he had been evaluated at an urgent care facility and was diagnosed with a presumed kidney stone, which he believed he had passed. He presented to the emergency room (ER) in late 2024 due to persistent abdominal pain and increasing abdominal distention. Associated symptoms included intermittent nausea, vomiting, early satiety, and mild lower urinary tract symptoms.

On presentation, vital signs were notable for tachycardia up to 130 beats per minute but were otherwise unremarkable. There was no documented fever. Physical examination revealed a large, palpable mass with associated tenderness in the left upper quadrant. Laboratory evaluation, including complete blood count and basic metabolic panel, was within normal limits. Urinalysis demonstrated ketones, protein, trace leukocyte esterase, 11–20 white blood cells per high‐power field, and 6–10 hyaline casts.

Computed tomography (CT) of the abdomen and pelvis demonstrated a markedly dilated left renal pelvis with near complete loss of renal parenchyma, significant mass effect on the spleen and great vessels, and a delayed nephrogram on the left (Figure 1a,b). No nephrolithiasis was noted in the left kidney. The contralateral right kidney and collecting system were normal. The left kidney measured 22.36 × 20.19 × 17.70 cm (height × width × length). The patient was discharged from the ER with close outpatient urology follow‐up for definitive management.

Figure 1(a) Axial CT scan with contrast performed prior to surgery. Demonstration of both kidneys noting the near absence of the renal parenchyma of the left kidney. Note the homogenous appearance of the fluid filled left renal pelvis. No nephrolithiasis was seen on final radiographic read.(b) Coronal CT scan with contrast performed prior to surgery. Demonstration of the massively enlarged left kidney exerting a mass effect in the peritoneal cavity, raising the left diaphragm and compressing the stomach, spleen, inferior vena cava, and aorta. Mass effect on the left sided abdominal wall can be seen as well. Note the septations within the left kidney as well as the homogenous appearance of the fluid filled left renal pelvis. No nephrolithiasis was seen on final radiographic read.

**Figure:**
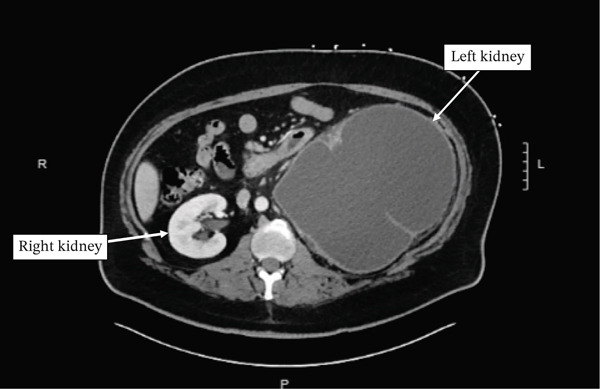
(a)

**Figure:**
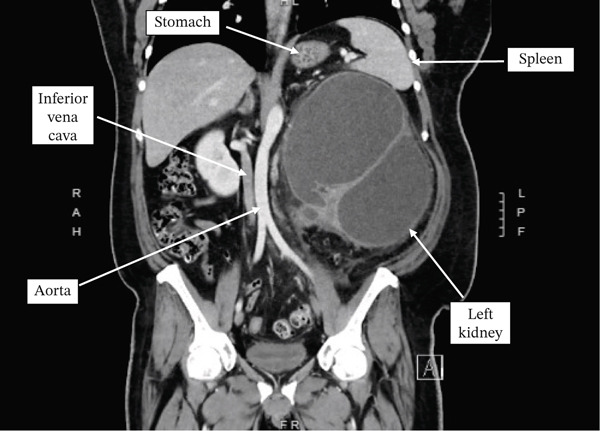
(b)

Surgical intervention was performed 4 weeks later. The patient underwent a transperitoneal laparoscopic robotic‐assisted left radical nephrectomy. Notably, upon initial Hasson entry, a markedly thinned renal pelvis was immediately encountered. This was sharply punctured, and approximately 2 L of purulent material were aspirated prior to docking the robot. Dissection of the kidney to the renal hilum was particularly challenging due to the absence of normal tissue planes secondary to chronic inflammation. The remainder of the procedure was completed without intraoperative complications. Total operative time was 210 min, including approximately 180 min of robotic console time.

**Figure 2:**
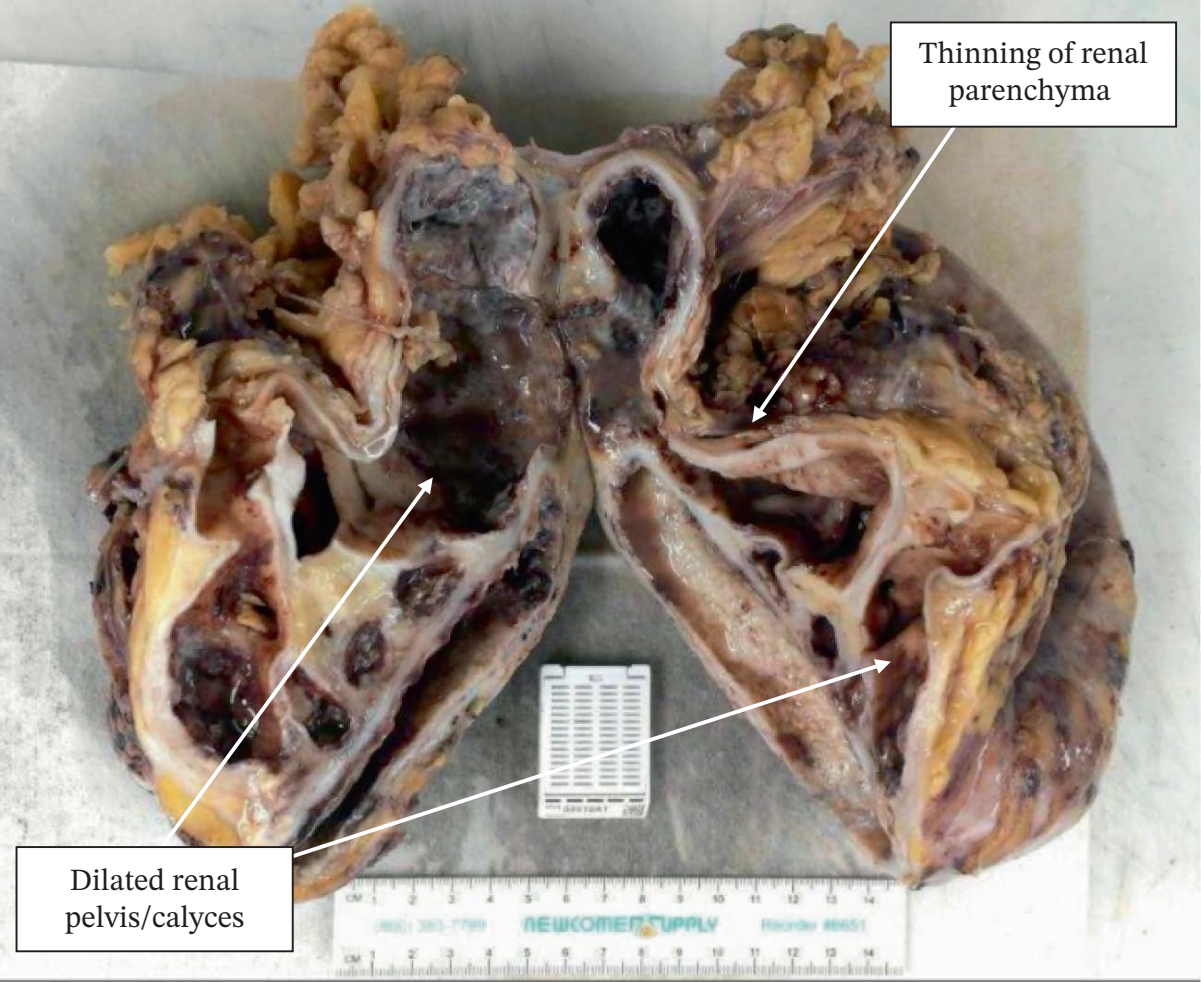
Bivalved gross specimen after laparoscopic robotic radical nephrectomy demonstrating the significantly dilated renal calyces. The near atrophy of the renal parenchyma can be appreciated. Also, note the infiltration of yellow tissue within the core of the specimen. Final pathological report indicating predominantly mixed inflammatory lining with granulation tissue; acute and chronic inflammation extend into the atrophic renal parenchyma and surrounding adipose tissue consistent with XGP. No evidence of neoplasm was identified.

Postoperatively, the patient recovered without complication. Gram stain of the aspirated fluid demonstrated a moderate number of polymorphonuclear leukocytes. Blood and urine cultures were obtained and were negative for aerobic, anaerobic, and fungal organisms. The patient was discharged on postoperative Day 4. Final pathologic examination revealed a cystically dilated renal pelvis and calyces with atrophic and inflamed renal parenchyma, consistent with XGP. The calyces were filled with pink–tan purulent material and contained areas of yellow necrosis (Figure 2). Lymph nodes included in the specimen were reactive, with no evidence of malignancy or active infection.

Postoperatively, the patient continued routine follow‐up with both his urology and primary care physician. At his 4‐month postoperative urology visit, he reported no concerns and his surgical wounds were well healed. At 6‐month follow‐up with his primary care physician, there were no reported complications or new concerns.

## 3. Discussion

XGP is a severe form of chronic pyelonephritis characterized by destruction of the renal parenchyma and replacement with granulomatous inflammatory tissue [1–3]. The condition was first described in the early 20th century by Schlagenhaufer [4]. In Western populations, XGP accounts for less than 1% of chronic pyelonephritis cases and demonstrates a predominance in female patients [3]. The underlying etiology is most often chronic urinary tract obstruction—commonly due to nephrolithiasis or UPJO—with superimposed infection. In this case, given the absence of definitive stone disease on prior imaging and no history of urologic surgery, UPJO was felt to be the underlying etiology. Typical clinical features include flank pain (69%), fever and chills (69%), and persistent bacteriuria (46%) [1–3]. A history of recurrent urinary tract infections or prior urologic instrumentation is frequently reported. Common causative organisms include *Escherichia coli*, *Enterococcus faecalis*, *Pseudomonas* species, *Proteus mirabilis*, and *Klebsiella* species [3, 5]. Although prolonged antibiotic therapy may be considered, surgical intervention has historically been the preferred definitive treatment. Management decisions are influenced by disease extent, ranging from involvement limited to the kidney (Stage I) to extension beyond the perinephric fat (Stage III) [4, 6]. Given its frequent radiographic and clinical overlap with renal malignancy, nephrectomy is often required to establish a definitive diagnosis.

More recent literature has supported the use of minimally invasive approaches, including robotic‐assisted laparoscopic nephrectomy, in the management of XGP. Several small‐sized to medium‐sized series have demonstrated that perioperative outcomes following robotic‐assisted laparoscopic nephrectomy for XGP are comparable to those of open surgery, without increased complication rates [7–9]. Additionally, robotic approaches offer well‐established benefits such as reduced blood loss, shorter hospital stay, improved cosmesis, and faster recovery. In the present case, the kidney was managed as an end stage UPJO without preoperative suspicion for Stage I XGP; however, transperitoneal robotic‐assisted laparoscopic radical nephrectomy proved to be an appropriate and successful approach based on the final pathology. Factors favoring a robotic approach included disease confined to the kidney, negative preoperative cultures, and the absence of systemic infection [7–9]. Furthermore, the patient′s morbid obesity posed a potential challenge for an open approach, which was mitigated by the ergonomic and visualization advantages of robotic surgery. Although our institution does not routinely perform retroperitoneal robotic nephrectomies, this approach may be reasonable for selected cases of Stage I XGP. In contrast, retroperitoneal access for Stage II or III disease would likely be technically challenging due to obliteration of tissue planes from chronic inflammation.

Emerging evidence has also explored nonoperative management strategies in select cases of segmental XGP. Chan et al. reported resolution of biopsy‐proven, nonseptic segmental XGP with culture‐directed antibiotic therapy alone, achieved with a 2‐week course and appropriate antimicrobial stewardship [6]. Similarly, Xie et al. demonstrated that prolonged preoperative antibiotic therapy in patients with diffuse XGP undergoing laparoscopic nephrectomy was associated with fewer and less severe postoperative complications [9]. However, given the extensive renal involvement and absence of active infection in our patient, neither antibiotic‐only management nor prolonged perioperative antibiotic therapy was appropriate. This case highlights the diagnostic variability of XGP and supports the role of robotic‐assisted laparoscopic nephrectomy as a safe and effective long‐term treatment option in carefully selected patients.

## 4. Conclusions

We demonstrate that a robotic‐assisted laparoscopic approach for Stage I diffuse XGP is feasible and consistent with emerging literature supporting minimally invasive management in appropriately selected patients. For Stage II and III disease, careful preoperative evaluation is warranted, as extensive inflammation and loss of normal tissue planes may favor an open surgical approach and consideration of prolonged preoperative antibiotic therapy.

## Funding

No funding was received for this manuscript.

## Ethics Statement

Institutional policy (CR HRP‐40) does not require additional informed consent for case reports that do not include patient identifiers. Written clinical consent obtained through the primary surgeon′s office (T.J.M.) includes authorization for the use of de‐identified clinical information in research projects related to the patient′s procedure.

## Conflicts of Interest

The authors declare no conflicts of interest.

## Data Availability

The data that support the findings of this study are available from the corresponding author upon reasonable request.
